# Critical Care–Specific vs General-Purpose Large Language Models in Emergency Intensive Care Unit Diagnosis: Single-Center Retrospective Paired Comparative Study

**DOI:** 10.2196/98026

**Published:** 2026-08-11

**Authors:** Lihong Zheng, Zeyu Lin, Xiaolu Liu, Zhao Fan, Zhong He, Junjie Xu, Lu Yin

**Affiliations:** 1 Department of Emergency Medicine Peking University Shenzhen Hospital Shenzhen, Guangdong China; 2 Shantou University Medical College Shantou, Guangdong China; 3 Shenzhen University Shenzhen, Guangdong China; 4 Clinical Research Institute Peking University Shenzhen Hospital Shenzhen, Guangdong China

**Keywords:** artificial intelligence–assisted diagnosis, critical illness, diagnostic accuracy, EICU, emergency intensive care unit, large language models, LLMs

## Abstract

**Background:**

The emergency intensive care unit (EICU) manages the most critically ill patients, where rapid and accurate diagnosis is essential yet challenging. Diagnostic error rates in this setting are more than twice as high as in general wards, with serious consequences for patient outcomes. Large language models (LLMs) have attracted growing interest as decision-support tools; however, direct comparative evidence between critical care–specialized and general-purpose LLMs across the admission-to-discharge diagnostic workflow remains limited.

**Objective:**

This study aimed to compare the top-1 diagnostic accuracy of a critical care–specific LLM (Qiyuan 3.0.1) with 3 general-purpose models (GPT‑5.1, DeepSeek V3.1, and Qwen3‑32B) for EICU diseases, providing evidence for intelligent tool selection.

**Methods:**

This single‑center retrospective paired study enrolled 184 consecutive EICU patients (April 2025-March 2026). Two standardized datasets were constructed: an initial dataset (first 24 hours of admission) and a final dataset (complete clinical course). All 4 models received identical zero‑shot prompts and generated diagnoses independently under masked conditions. The gold standard was the consensus diagnosis by 3 senior intensivists (>10 years’ EICU experience; Fleiss κ=0.82). The primary end point was final‑stage top-1 accuracy; secondary end points were initial‑stage top-1 accuracy and the number of correctly matched diagnoses among the first 3 outputs at the final stage. Overall comparisons used Cochran Q test, followed by paired McNemar tests with Bonferroni correction; intergroup differences for top-3 counts were assessed by Friedman rank sum test.

**Results:**

Final-stage top-1 accuracy varied significantly across models (Cochran Q=20.32; *P*<.001): Qiyuan 3.0.1 reached 64.1% (118/184), followed by GPT-5.1 (109/184, 59.2%), DeepSeek V3.1 (105/184, 57.1%), and Qwen3-32B (95/184, 51.6%). Corrected pairwise comparisons (α=.0083) confirmed Qiyuan 3.0.1, GPT-5.1, and DeepSeek all outperformed Qwen3-32B significantly (all adjusted *P*<.008), while no statistical gaps were detected between Qiyuan 3.0.1 and the 2 top-performing general models. Though overall initial-stage accuracy differed significantly (Cochran Q=13.87; *P*<.001), no pairwise comparisons yielded significant results after correction. All models shared a median of 2 (IQR 1.0-2.0) correct top-3 diagnoses with no intergroup disparity (Friedman *χ*^2^_3_=3.34; *P*=.34). Notably, all models’ top-1 accuracy stayed below 70%, and stratified analysis revealed heterogeneous performance across core EICU diseases including sepsis, severe pneumonia, and gastrointestinal bleeding.

**Conclusions:**

Under the specific data conditions of this study, the critical care–specialized Qiyuan 3.0.1 performed comparably to leading general‑purpose LLMs (GPT‑5.1 and DeepSeek V3.1), supporting its potential for further specialty‑oriented exploration. Nevertheless, absolute accuracy below 70% precludes its direct deployment as an independent diagnostic standard. Bridging the gap from preliminary evaluation to clinical translation requires multicenter external validation, prospective human‑machine collaboration trials, and deeper model optimization—including sustained fine‑tuning on critical care corpora, transparent reasoning pathway design, and systematic safety boundary assessment.

## Introduction

The emergency intensive care unit (EICU) is the core medical unit for the treatment of acutely and critically ill patients. Admitted patients generally present with clinical characteristics of acute onset, rapid disease progression, complex pathophysiological mechanisms, and frequent multiple organ dysfunction, which place extremely high demands on the rapid and accurate diagnostic capabilities of clinicians [1]. Previous studies have shown that the diagnostic error rate in intensive care units (ICUs) is as high as 23%, more than twice that of general inpatient wards; the rate of substantial discrepancies between the initial diagnosis made by emergency physicians and the final diagnosis at patient discharge can reach 16%, which can lead to delayed treatment, adverse prognosis, and even patient death in severe cases [2-5]. In a clinical environment with high workload and high uncertainty, EICU physicians face enormous cognitive load and decision-making pressure, which further increases the risk of diagnostic errors [6]. Therefore, there is an urgent need for effective auxiliary tools to optimize the EICU diagnostic process, reduce diagnostic error rates, and improve clinical efficiency. The rapid development of Generative AI has provided important support for innovative changes in the medical field. As a core branch of Generative AI, large language models (LLMs) have shown application potential in multiple medical scenarios such as medical education, clinical document generation, and auxiliary diagnosis, relying on their powerful natural language understanding, multisource information integration, and logical reasoning capabilities [7]. Previous studies have confirmed that general-purpose LLMs without specialized fine-tuning can pass the United States Medical Licensing Examination and the Chinese Medical Licensing Examination, and provide standardized diagnosis and treatment recommendations for common diseases [8-10]. Meanwhile, LLMs can generate standardized admission notes and discharge summaries based on clinical data, effectively reducing the documentation burden of clinicians [11,12]. They provide an innovative path for optimizing clinical workflows and improving service efficiency, thus promoting the transformation of the medical field toward intelligence and precision, and laying a foundation for their application in the field of critical care.

Theoretically, LLMs can alleviate the cognitive load of clinicians and reduce the risk of diagnostic errors in the EICU by rapidly integrating massive clinical data of patients and mining potential diagnostic clues. However, existing studies still have obvious limitations. First, most existing studies on the diagnostic performance of LLMs focus on general wards, outpatient clinics, or conventional emergency department settings, and there is a serious lack of specialized research targeting the high-acuity, high-complexity, and time-sensitive diagnostic scenarios of the EICU [13-16]. Second, most existing studies only evaluate the diagnostic performance of general-purpose LLMs, and there is a scarcity of paired comparative studies between critical care-specific fine-tuned LLMs and mainstream general-purpose LLMs in the EICU setting. Third, most studies only evaluate the final diagnostic performance of models based on complete course data and do not simulate the progressive diagnostic process from initial admission assessment to final diagnosis, which is insufficiently aligned with real clinical practice [17-20]. These issues highlight the necessity of systematically evaluating the diagnostic performance of a critical care-specific LLM (Qiyuan) and mainstream general-purpose models (GPT, DeepSeek, and Qwen) in the face of complex diseases. This study adopted a 2-stage dataset design (initial diagnosis and final diagnosis) to simulate the progressive clinical decision-making process from admission to diagnosis confirmation, so as to more realistically reflect the application value of LLMs in the actual workflow of the EICU.

As an offline diagnostic accuracy benchmark, it represents a foundational step in the evaluation pipeline for LLM-based clinical decision support tools, which ultimately aim to be deployed through web-based or integrated EHR platforms in real-world EICU settings.

Based on this, this study systematically evaluated the diagnostic performance of the critical care–specific LLM Qiyuan 3.0.1 and 3 mainstream general-purpose LLMs (GPT-5.1, DeepSeek V3.1, and Qwen3-32B) in the initial and final diagnosis stages of EICU patients through a single-center retrospective paired design. This study aimed to address 3 key scientific questions: first, whether the critical care-specific LLM achieves superior diagnostic accuracy over general-purpose counterparts; second, how the diagnostic performance of different LLMs changes from the initial to the final diagnosis stage; and third, to clarify the application potential and limitations of LLMs in EICU auxiliary diagnosis, thereby providing an evidence base for model optimization and clinical translation.

## Methods

### Study Design

**Figure 1:**
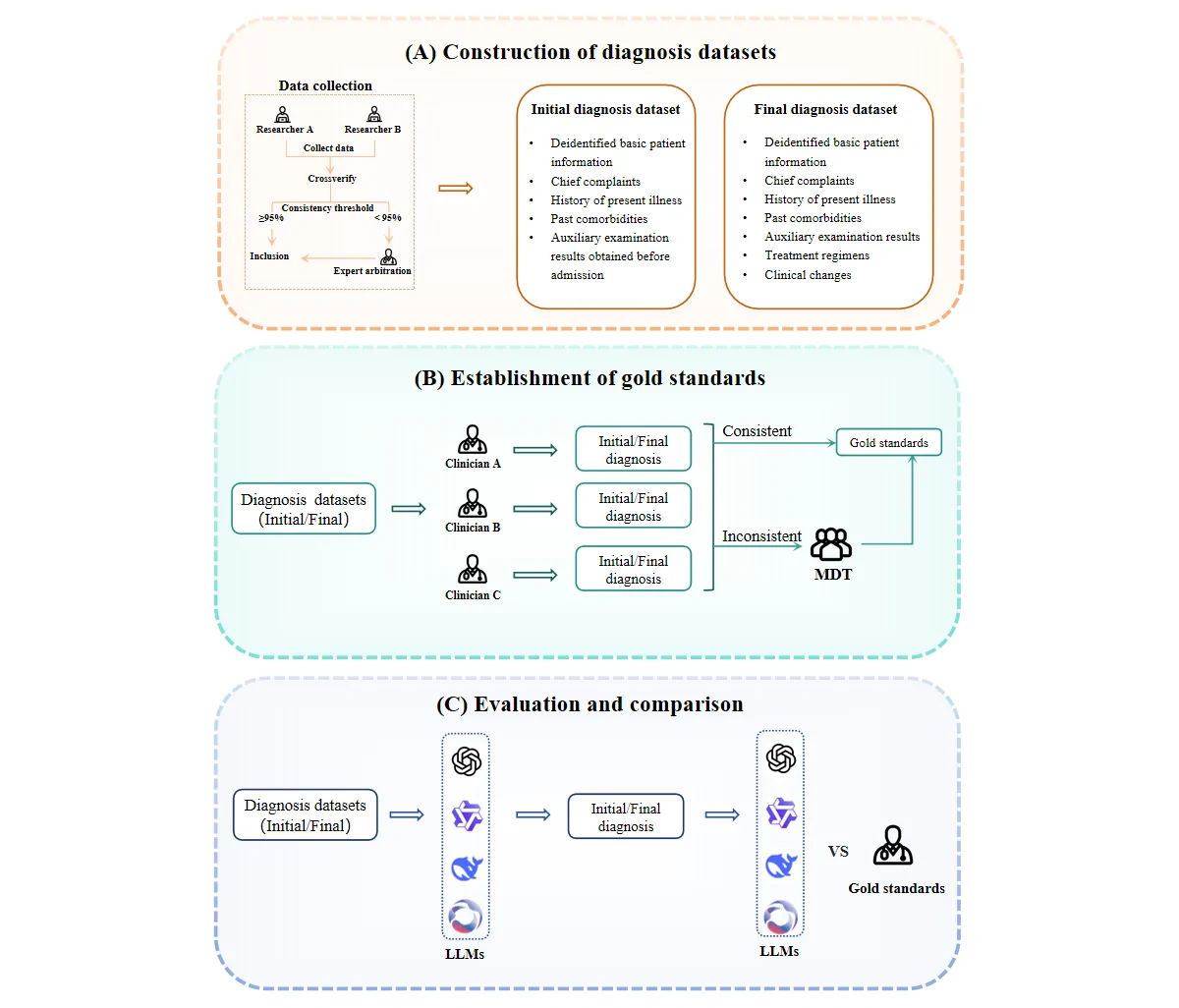
Overview of the study workflow. This single-center retrospective paired diagnostic accuracy study was conducted in the emergency intensive care unit of Peking University Shenzhen Hospital (April 2025-March 2026) and included 184 consecutive patients. (A) Construction of 2 standardized datasets (initial 24-hour data and full course final data). (B) Establishment of the gold standard by 3 senior intensivists (Fleiss κ=0.82), with multidisciplinary team (MDT) consensus for discrepant cases. (C) Independent diagnostic evaluation of 4 large language models (LLMs; Qiyuan 3.0.1, GPT 5.1, DeepSeek V3.1, and Qwen3 32B) under masked conditions, compared against the gold standard.

This was a single-center retrospective paired diagnostic accuracy study conducted in the EICU of Peking University Shenzhen Hospital, consecutively enrolling eligible critically ill patients admitted from April 2025 to March 2026. All model outputs were evaluated under blinded conditions: the 3 intensivists who established the gold standard were masked to the diagnostic outputs of all LLMs, and the researchers performing statistical analyses were blinded to the concordance between model outputs and the gold standard until the final dataset was locked. The study was reported in accordance with the Transparent Reporting of a Multivariable Model for Individual Prognosis or Diagnosis for Large Language Models (TRIPOD-LLM) reporting guideline for LLM predictive research [21]; the completed TRIPOD-LLM checklist is provided in Multimedia Appendix 1 [21]. The clinical data of all included patients were strictly deidentified and anonymized before use to ensure that the personal identity of patients could not be traced. The application of LLMs was strictly limited to research data analysis and did not interfere with any clinical diagnosis and treatment decision-making process, nor did it affect the clinical diagnosis and treatment outcomes of patients. Figure 1 provides an overview of the study workflow.

### Ethics Approval

This study protocol obtained formal ethical clearance from the Medical Ethics Committee of Peking University Shenzhen Hospital (approval number 2026-029 [Research]-01) prior to data extraction and model analysis, fully complying with the institutional human research oversight requirements, the World Medical Association Declaration of Helsinki, and the Personal Information Protection Law of the People’s Republic of China [21].

### Study Participants and Inclusion and Exclusion Criteria

This study retrospectively and consecutively enrolled patients admitted to the EICU of Peking University Shenzhen Hospital from April 2025 to March 2026. The inclusion criteria were as follows: patients were included if they were aged 16 years or older, had an EICU stay longer than 24 hours, possessed complete medical records covering both the initial 24-hour data and the full clinical course, and had a definitive final clinical diagnosis.

Exclusion criteria comprised death, discharge against medical advice, or transfer within 24 hours of admission; severe missing core clinical data precluding standardized dataset construction; end‑stage disease receiving palliative care without a clear diagnostic goal; and cases with unresolved diagnostic disputes after multidisciplinary team (MDT) consultation.

**Figure 2:**
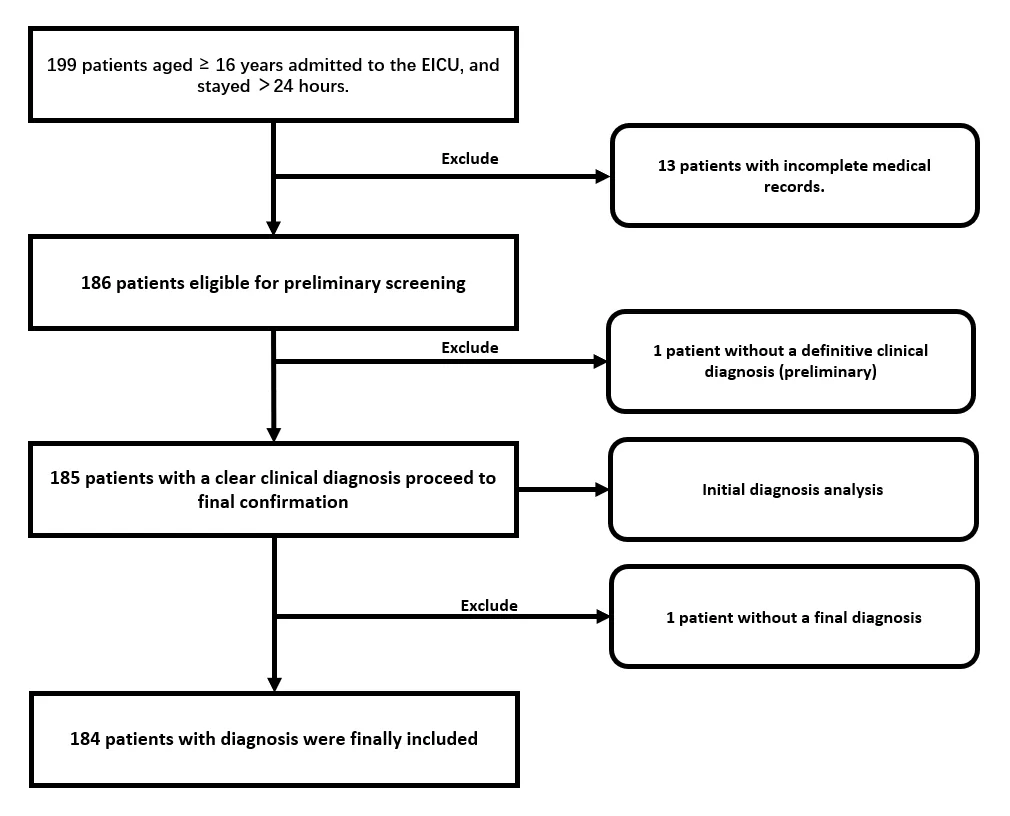
Flowchart of inclusion and exclusion of research participants. This single-center retrospective study enrolled consecutive patients aged ≥16 years admitted to the emergency intensive care unit (EICU) of Peking University Shenzhen Hospital from April 2025 to March 2026 with an EICU stay of >24 hours. After applying inclusion and exclusion criteria, 184 of 199 screened patients were included for large language model diagnostic evaluation.

A total of 199 consecutive patients meeting the time range were initially screened in this study. After screening according to the above criteria, a total of 184 complete cases were finally included, covering common and high-incidence diseases in the EICU (sepsis, acute respiratory distress syndrome, cardiovascular emergencies, neurological emergencies, etc). The screening flow of study participants is shown in Figure 2.

### Sample Size Calculation

This study adopted a paired design, with the primary end point being the top-1 accuracy in the final diagnosis stage. The core statistical analysis was paired pairwise comparisons among the 4 models, and the sample size was calculated using the paired McNemar test. Based on the results of our team’s preexperiment (30 EICU cases), the top-1 accuracy of Qiyuan 3.0.1 in the final diagnosis was 72%, and that of the best-performing general-purpose model GPT-5.1 was 54%, with an absolute difference of 18%, which was set as the minimum clinically significant difference to be detected in the EICU clinical setting. The proportion of discordant pairs was 36.7% (11/30), of which the proportion of cases with correct diagnosis by Qiyuan 3.0.1 but incorrect by the control model was 23.3% (7/30), and the proportion of cases with incorrect diagnosis by Qiyuan 3.0.1 but correct by the control model was 13.3% (4/30).

A 2-tailed significance level of α=.05 was set, with a target power (1-*β*) of 0.80. Considering that a total of 6 pairwise comparisons were required among the 4 models, the Bonferroni method was used to correct for type I error, with an adjusted significance level of α’=.05/6≈.0083. The calculation was performed using the “Paired Proportions (McNemar Test)” module of PASS 15 software (NCSS, LLC), and the minimum required sample size was 156 cases. Considering a 10% case dropout and data missing rate, the planned sample size was 172 cases.

A total of 184 cases were finally included in this study, which exceeded the preset minimum sample size. Post hoc power analysis based on the actual data of the final study showed that the power of this study was 86.2%, which exceeded the preset 80%, confirming that the sample size was sufficient to detect the preset clinical difference with a controllable risk of false negative results.

### Dataset Construction and Prompt Engineering

#### Construction of Standardized Diagnostic Datasets

To simulate the progressive diagnostic process from initial assessment to final confirmation in clinical practice, we constructed 2 standardized diagnostic datasets with strictly defined time windows and completely standardized formats. All datasets were independently collected and cross‑checked by 2 researchers, with a data consistency threshold of ≥95%. Discrepancies were arbitrated by senior EICU physicians to ensure that the case data fed into the 4 models were identical in format. The first dataset, termed the initial diagnosis dataset, encompassed all clinical data obtained within 24 hours after EICU admission, with the time window starting precisely at the moment of patient transfer to the EICU. All examinations, laboratory tests, and treatment records generated after the 24‑hour limit were rigorously excluded to mirror the emergency decision‑making scenario typical of the early admission phase. This dataset included standardized fields such as basic patient information (age, sex, past medical history, and allergy history), chief complaint, history of present illness, physical examination on admission, and auxiliary investigations performed in the emergency department before admission and within the first 24 hours of EICU stay—comprising complete blood count, biochemistry, coagulation function, blood gas analysis, infection markers, electrocardiogram, and imaging studies—as well as medication orders and vital sign recordings during that initial 24‑hour period. The second dataset, designated as the final diagnosis dataset, built upon the initial dataset by supplementing the complete longitudinal clinical data from EICU admission to hospital discharge, which included subsequent confirmatory test results (eg, pathology reports, surgical records, and metagenomic next‑generation sequencing results for pathogenic microorganisms), specialist consultation notes, and comprehensive records of treatment outcomes. Crucially, any text that directly stated the definitive clinical diagnosis—such as the discharge diagnosis section of the discharge summary or the clinical diagnosis entries on pathology request forms—was deliberately removed to ensure that the models independently derived their diagnoses solely from the raw clinical data, thereby simulating the real‑world final diagnostic process undertaken by clinicians after they have acquired the full clinical picture.

To further minimize diagnostic information leakage, we applied a systematic protocol beyond simply removing discharge diagnosis sections. All text fields containing unequivocal diagnostic labels—such as “final diagnosis,” “clinical impression,” or “pathological diagnosis” headers—were redacted from the raw clinical documents. However, we deliberately retained the descriptive raw data contained in these reports (eg, pathology morphology descriptions, microbiological culture and metagenomic sequencing results, and surgical procedure notes) because these represent the same type of raw clinical evidence that EICU physicians use during diagnostic workup, rather than preinterpreted diagnostic conclusions. This approach reflects real-world clinical reasoning: clinicians formulate differential diagnoses from raw laboratory, imaging, and pathological findings, not from prewritten diagnostic summaries. While we acknowledge that some auxiliary reports (eg, pathology morphology or metagenomic sequencing results) carry strong diagnostic implications, these are also part of the standard clinical information available to EICU physicians during routine care; excluding them would have artificially constrained the models’ diagnostic inputs and reduced the external validity of the evaluation. The key distinction is that we removed explicitly concluded diagnostic labels, as well as treatment choices and treatment outcome records, while retaining the descriptive data that clinicians themselves use to reach diagnostic conclusions.

#### Standardized Prompt Strategy

To control the impact of prompt differences on model output, a unified Chinese prompt template was adopted for all 4 LLMs in this study. A strict zero-shot learning strategy was used, which only relied on the model’s own parameterized knowledge to complete the diagnosis without additional few-shot examples or specialized knowledge injection [22,23].

We deliberately selected zero-shot prompting for 2 primary reasons. First, zero-shot prompting without iterative optimization better simulates real-world EICU deployment scenarios, where clinicians expect AI tools to function reliably in a “plug-and-play” manner without extensive prompt engineering. Second, by applying an identical prompt to all 4 models, we ensured that prompt-related variability was held constant across comparators, allowing a fair assessment of each model’s out-of-the-box diagnostic performance under identical input conditions. The prompt template is presented in Textbox 1.

**Prompt template.**

You are an experienced specialist in the intensive care unit, with profound clinical attainments in acute and critical care medicine, keen diagnostic ability for acute diseases, and rigorous clinical logical reasoning ability. Your task is to conduct a comprehensive analysis and evaluation based on the complete clinical data of the patient provided below, derive the most probable and accurate diagnosis, and attach concise and rigorous core diagnostic basis.

The models were instructed to generate diagnoses strictly based on the provided clinical data, without adding any external information; to output the primary diagnosis first followed by secondary diagnoses in order of clinical priority; and to align their diagnostic terms as closely as possible with the *International Classification of Diseases, 10th Revision* (*ICD-10*).

The patient’s clinical data are as follows: [Embedded standardized case dataset].

### LLM Configuration and Diagnosis Implementation

Four versions of LLMs were used in this study, including 1 critical care–specific LLM (Qiyuan 3.0.1) and 3 mainstream general-purpose LLMs (GPT-5.1, DeepSeek V3.1, and Qwen3-32B). The detailed configuration of the models is shown in Table 1 and Table S2 in Multimedia Appendix 2. To control the impact of generation randomness on diagnostic results, all models were configured with the following generation parameters: temperature=0.0 (deterministic output), top-p=1.0, top-k=0(disabled), Max tokens=20000, and Random seed=42. All models used the same reasoning mode (enable_thinking=true). The exact model versions were as follows: GPT-5.1 (release 2025.11.12) via the Microsoft Azure OpenAI Service application programming interface (API); DeepSeek V3.1 (release 2025.08.21) via the Volcengine API; Qwen3-32B (release 2025.04.28) and Qiyuan 3.0.1 (release 2025.08.01) deployed locally. Parameter support differs across API and locally deployed platforms. Temperature, top-p, top-k, and Max tokens were supported and effective for all models. The random seed parameter was supported for locally deployed models (Qwen3-32B and Qiyuan 3.0.1) and for the Azure OpenAI API (GPT-5.1) but could not be verified for the Volcengine API (DeepSeek V3.1), as seed control is not uniformly exposed across all API providers. Full details, including API end point identifiers and platform version numbers, are provided in Table S2 in Multimedia Appendix 2.

**Table 1:** Details of included large language models.

Model	Developer	Version (release date)	Deployment platform	Parameter scale
GPT	OpenAI	GPT-5.1 (2025.11.12)	Microsoft Azure API^a^	Undisclosed
DeepSeek	DeepSeek	DeepSeekV3.1 (2025.8.21)	Volcengine API	685B
Qwen	Alibaba	Qwen3-32B (2025.4.28)	On Premise	32B
Qiyuan	Mindray	Qiyuan 3.0.1 (2025.8.1)	On Premise	32B

^a^API: application programming interface.

To mitigate this limitation, we randomly selected 10 cases (approximately 5% of the cohort) and repeated each model inference 3 times under the temperature=0.0 setting; all repeated queries produced identical outputs for all 4 models, confirming deterministic behavior at the time of evaluation. For the main evaluation, each of the 184 cases was run once per model. Nevertheless, we acknowledge that residual nondeterminism at the infrastructure level—such as hardware-level floating-point variation, model serving batching, or unannounced model updates—may introduce variability that is not fully controlled by temperature settings.

We implemented 3 masking and independence measures. First, the 3 senior physicians who established the gold standard were masked to the diagnostic outputs of all LLMs throughout the study. Second, all LLMs performed diagnoses independently without any information exchange between models. Third, the researchers responsible for data entry and statistical analysis remained masked to the gold standard diagnoses until the analysis was completed.

Furthermore, to ensure transparency and reproducibility regarding the models used, all model versions cited in this study—GPT-5.1, DeepSeek V3.1, Qwen3-32B, and Qiyuan 3.0.1—were publicly available at the time of analysis and could be accessed via their official APIs or local deployment channels. The version numbers and release dates provided in the text correspond to the officially stable releases during the analysis period, not to hypothetical or unreleased future versions. In this study, GPT-5.1 was accessed through the Microsoft Azure OpenAI Service API, DeepSeek V3.1 through the Volcengine API, and Qwen3-32B and Qiyuan 3.0.1 were deployed locally using vendor-provided, validated model weight files. Detailed model parameters and generation configurations are provided in Table 1 and the main text. For additional technical specifications, readers are referred to the official documentation of each provider (Table S2 in Multimedia Appendix 2). Of note, Qiyuan 3.0.1 is a proprietary model developed by Mindray that was built upon the open‑source Qwen3-32B architecture, with additional domain‑specific fine‑tuning and reinforcement learning optimization (Table S2 in Multimedia Appendix 2). This relationship is important to highlight because the comparison between Qiyuan 3.0.1 and Qwen3‑32B—both of which have a 32B parameter scale and were deployed locally—represents the most directly informative, though noncausal, contrast between a specialty‑adapted model and its general‑purpose base in this study.

### Gold Standard Establishment

The gold standard diagnosis in this study was developed by 3 senior intensive care specialists with more than 10 years of EICU clinical working experience and a title of associate chief physician or above. All experts were blinded to the diagnostic results of the LLMs throughout the study. Based on the deidentified complete clinical data, they independently developed the initial and final diagnoses for each case, with the diagnosis list including 1 primary diagnosis and multiple secondary diagnoses (concomitant diseases/complications) [24]. Cases with completely consistent diagnoses from the 3 physicians directly used the consensus diagnosis as the gold standard; cases with diagnostic discrepancies were submitted to MDT consultation consisting of specialists in critical care medicine, emergency medicine, and corresponding specialties, and the final consensus diagnosis reached by the collective was used as the final gold standard.

Fleiss κ test was used to evaluate the interrater reliability of the independent diagnoses by the 3 physicians. The results showed that the 3 physicians had good consistency in the independent judgment of the primary diagnosis (Fleiss κ=0.82, 95%CI 0.76-0.88; *P*<.001), confirming that the gold standard establishment had good reliability and stability.

For the initial‑diagnosis gold standard, the 3 physicians were explicitly instructed to base their independent diagnostic judgments solely on the clinical data available within the first 24 hours of EICU admission—the same information available to the models in the initial stage. For the final‑diagnosis gold standard, the physicians used the complete longitudinal dataset up to hospital discharge. This design ensured information parity between the gold standard and model inputs at each stage.

Fleiss κ test was used to evaluate the interrater reliability of the independent diagnoses by the 3 physicians. For the primary diagnosis, the 3 physicians showed good consistency (Fleiss κ=0.82, 95% CI 0.76-0.88; *P*<.001). For secondary diagnoses, the agreement was also substantial (Fleiss κ=0.79, 95% CI 0.72-0.85; *P*<.001). These results confirm that the gold standard establishment had good reliability and stability for both primary and secondary diagnostic components, supporting the validity of the top-3 end point, which relies on matching both primary and secondary diagnoses.

### Study End Points and Evaluation Rules

The primary end point of this study was the top-1 accuracy in the final diagnosis stage, and the secondary end points included the top-1 accuracy in the initial diagnosis stage and the number of correct diagnoses in the top-3 outputs in the final diagnosis stage. The specific definitions and evaluation rules are as follows:

Model outputs were parsed by extracting the first listed diagnosis as the primary diagnosis and the subsequent diagnoses as secondary diagnoses, in the order generated by the model. Diagnoses were identified by scanning the output text for diagnostic statements preceding or following the primary diagnostic label.

The top-1 accuracy in the final diagnosis stage was defined as the proportion of cases where the first primary diagnosis output by the model completely matched the primary diagnosis of the gold standard. The matching rule was that the clinical connotation of the 2 was consistent, and the *International Classification of Diseases, Tenth Revision* (*ICD‑10*) coding was completely matched to the subcategory level. If the diagnosis name output by the model was not clearly specified to the subcategory level but the clinical semantics were equivalent to the gold standard diagnosis (eg, “acute respiratory failure” and the gold standard “acute hypoxic respiratory failure”), 2 researchers independently ruled on whether it was judged as a match, and discrepancies were arbitrated by a third senior physician. The frequency of soft‑match adjudication, its distribution across models, and interrater agreement between the 2 adjudicators were assessed. A sensitivity analysis comparing the main results under a strict *ICD‑10* matching rule vs the semantic soft‑match rule adopted in this study is provided in Table S1b in Multimedia Appendix 2. Furthermore, to evaluate the interrater reliability of the top-1 match adjudication (match vs no match) in the final diagnosis stage, unweighted Cohen κ was calculated, yielding a κ of 0.86 (95% CI 0.81-0.91).

The definition and matching rules of the top-1 accuracy in the initial diagnosis stage were the same as those of the primary end point, which was calculated based solely on the model outputs from the initial diagnosis dataset. To evaluate the interrater reliability of the binary correctness classification (correct vs incorrect) for the initial diagnosis stage, unweighted Cohen κ was calculated, yielding a κ of 0.92 (95% CI 0.89-0.95), indicating almost perfect reproducibility of the evaluation process for the initial diagnostic outputs.

The number of correct diagnoses in the top-3 outputs in the final diagnosis stage was defined as the count of diagnoses (range 0-3) within the first 3 diagnoses output by each model that matched the gold standard diagnosis list (comprising 1 primary and 2 secondary diagnoses), with diagnoses prioritized in the order generated by the model. The same matching rule applied: a diagnosis was considered correct if its clinical connotation aligned with the corresponding diagnosis in the gold standard list and its *ICD-10* code matched at the subcategory level. Given the ordinal nature of this 0-3 scale, interrater reliability for the top-3 count scoring was assessed using quadratic weighted Cohen κ, yielding a weighted κ of 0.95 (95% CI 0.93-0.96), indicating almost perfect agreement between the 2 adjudicators on the combined primary-plus-secondary diagnosis matching process. A summary of interrater reliability across all evaluation stages is provided in Table S1a in Multimedia Appendix 2.

### Statistical Analysis

#### Overview

All statistical analyses were 2-tailed, with a significance level of α=.05. Statistical analysis was performed using R software (version 4.4.3; R Foundation for Statistical Computing; stats package, coin package, and irr package), and graphs were plotted using GraphPad Prism software (version 10; GraphPad Software Inc).

#### Baseline Data Statistics

Normally distributed measurement data were expressed as mean (SD), and nonnormally distributed measurement data were expressed as median (IQR). Count data were expressed as number of cases (percentage; n [%]).

#### Statistics for Top-1 Accuracy

Diagnostic accuracy was reported as a percentage with 95% CI, and the CI was calculated using the Clopper-Pearson exact method. For the paired binary outcomes of the 4 models in the same diagnosis stage, the Cochran Q test was used for the overall comparison of differences among multiple groups. If the overall test showed a statistically significant difference, post hoc pairwise comparisons were performed using the paired McNemar test with Bonferroni correction for type I error. For 6 pairwise comparisons among the 4 models, the adjusted significance level was set to α’=.05/6≈.0083.

The paired McNemar test was used to compare the difference in top-1 accuracy between the initial and final diagnosis stages for each model, to evaluate the impact of increased clinical information on the diagnostic performance of the model.

#### Statistics for the Number of Correct Top-3 Diagnoses

This indicator was repeated-measurement discrete count data corresponding to the 4 models for the same case. The overall comparison of differences among the 4 models was performed using the Friedman test. Descriptive statistics were presented as median (IQR), and the 95% CI for the median was estimated using the nonparametric Bootstrap method (1000 resamples). The distribution of this indicator across the 4 models was visualized via a stacked bar chart.

## Results

### Baseline Characteristics of the Study Population

A total of 184 EICU patients were finally included in this study, including 118 (64.1%) male and 66 (35.9%) female individuals, with a median age of 65 (IQR 53-76) years. The median length of EICU stay was 4.6 (IQR 2.47-8.95) days, the median Acute Physiology and Chronic Health Evaluation II (APACHE II) score on admission was 18.5 (IQR 14-24) points, and the in-hospital mortality rate was 10.3% (19/184). Table 2 summarizes the baseline characteristics of the study population.

**Table 2:** Baseline characteristics of the study population (N=184).a

Characteristic	Value
Age (years), median (IQR)	65 (53-76)
Male sex, n (%)	118 (64.1)
EICU^b^ stay (days), median (IQR)	4.6 (2.47-8.95)
APACHE II^c^ score on admission, median (IQR)	18.5 (14-24)
In‑hospital mortality, n (%)	19 (10.3)

^a^Data are from a single‑center retrospective paired study of 184 consecutive EICU patients (Peking University Shenzhen Hospital, April 2025-March 2026).

^b^EICU: emergency intensive care unit.

^c^APACHE II: Acute Physiology and Chronic Health Evaluation II.

The distribution of the main disease spectrum of patients was as follows: sepsis/septic shock in 39 (21.2%) cases, severe pneumonia in 36 (19.6%) cases, acute coronary syndrome/acute heart failure in 24 (13%) cases, massive gastrointestinal bleeding in 17 (9.2%) cases, acute stroke/central nervous system infection in 12 (6.5%) cases, and other diseases in 56 (30.4%) cases.

### Primary End Point: Top-1 Accuracy in the Final Diagnosis Stage

In the final diagnosis stage, the overall difference in top-1 accuracy among the 4 models was statistically significant (Cochran Q=20.32, df=3; *P*<.001). Qiyuan 3.0.1 had the highest top-1 accuracy at 64.1% (118/184, 95% CI 56.8%-71.0%), followed by GPT‑5.1 at 59.2% (109/184, 95% CI 51.8%-66.4%), DeepSeek V3.1 at 57.1% (105/184, 95% CI 49.6%-64.3%), and Qwen3‑32B had the lowest accuracy at 51.6% (95/184, 95% CI 44.3%-59.0%).

**Figure 3:**
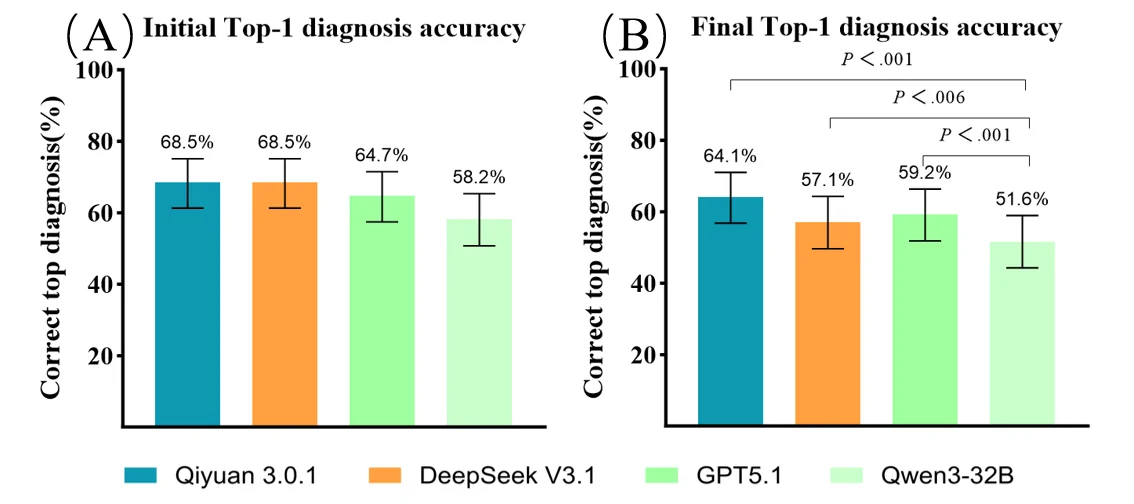
Comparison of top-1 accuracy of the 4 models in the initial and final diagnosis stages. Data are from a single-center retrospective paired study of 184 emergency intensive care unit patients (Peking University Shenzhen Hospital, April 2025-March 2026). Error bars represent 95% Clopper-Pearson exact CIs. Overall comparison was performed using the Cochran Q test, with pairwise comparisons using the McNemar test and Bonferroni correction (α=.0083). (A) Initial stage (24-hour data). (B) Final stage (full course data).

Post hoc pairwise comparisons (McNemar’s test, adjusted α=.0083) showed that Qiyuan 3.0.1, GPT‑5.1, and DeepSeek V3.1 all significantly outperformed Qwen3‑32B (adjusted *P*<.001, <.001, and .006, respectively; all <.008). No other pairwise comparisons reached the adjusted significance threshold (all adjusted *P*>.008). These results are presented in Figure 3B.

Among all cases correctly diagnosed by each model, the proportion that were confirmed via soft-match (rather than strict *ICD-10* subcategory matching) was as follows: 71/118 (60.2%) for Qiyuan 3.0.1; 78/109 (71.6%) for GPT-5.1; 68/105 (64.8%) for DeepSeek V3.1; and 55/95 (57.9%) for Qwen3-32B. A sensitivity analysis comparing the top-1 accuracy under strict *ICD-10* matching vs the semantic soft-match rule confirmed that the relative model rankings remained unchanged under both matching rules (Table S1b in Multimedia Appendix 2).

### Secondary End Point 1: Top-1 Accuracy in the Initial Diagnosis Stage

#### Overview

In the initial diagnosis stage, the overall difference in top‑1 accuracy among the 4 models was statistically significant (Cochran Q=13.87, df=3; *P*<.001). Qiyuan 3.0.1 and DeepSeek V3.1 had the highest top‑1 accuracy, both at 68.5% (126/184, 95% CI 61.3%-75.1%), followed by GPT‑5.1 at 64.7% (119/184, 95% CI 57.4%-71.5%), and Qwen3‑32B had the lowest accuracy at 58.2% (107/184, 95% CI 50.7%-65.3%).

Post hoc pairwise comparisons did not reveal any statistically significant differences after Bonferroni correction. Notably, the comparison between Qiyuan 3.0.1 and Qwen3-32B yielded an adjusted *P* value of .08, which substantially exceeded the Bonferroni-corrected threshold of .008; the closest comparison was Qwen3-32B vs DeepSeek V3.1 (adjusted *P*=.03). All other pairwise comparisons yielded adjusted *P*>.99. These results are presented in Figure 3A.

#### Error Pattern Analysis

To characterize the nature of diagnostic errors beyond overall accuracy, we performed a descriptive categorization of all incorrect diagnoses according to predefined diagnostic distance tiers (Table S3a in Multimedia Appendix 2). This classification is intended for descriptive characterization of error patterns and does not imply validated grading of clinical harm. A Tier 1 error (correct diagnosis present among the top-3 outputs but not in first place) should not be equated with clinically minor impact without case-level safety adjudication. For Qiyuan 3.0.1, a total of 66 errors were recorded, among which 18 (27.3%) were classified as Tier 1 (correct diagnosis present in the top‑3 outputs but not in first place), 6 (9.1%) as Tier 2 (correct disease category but wrong subcategory), and 42 (63.6%) as Tier 3 (complete deviation in etiology or organ system). For GPT‑5.1, 75 errors were observed, with 12 (16%) Tier 1, 15 (20%) Tier 2, and 48 (64%) Tier 3 errors. For DeepSeek V3.1, 79 errors were identified, comprising 7 (8.9%) Tier 1, 16 (20.3%) Tier 2, and 56 (70.9%) Tier 3 errors. For Qwen3‑32B, 89 errors were noted, including 10 (11.2%) Tier 1, 36 (40.5%) Tier 2, and 43 (48.3%) Tier 3 errors. A further qualitative breakdown of the Tier 3 complete‑deviation errors is provided in Table S3b in Multimedia Appendix 2.

#### Comparison of Diagnostic Performance Between Initial and Final Stages

To evaluate whether the accumulation of full clinical course data improved diagnostic accuracy, we compared the top-1 accuracy of each model between the initial (24-hour data) and final (complete data) stages using the paired McNemar test. Although numerical declines were observed for all 4 models from the initial to the final stage, none of these within-model differences reached statistical significance: Qiyuan 3.0.1 (68.5% vs 64.1%; McNemar *χ*^2^_3_=0.65; *P*=.42), GPT-5.1 (64.7% vs 59.2%; *χ*^2^_3_=1.03; *P*=.31), DeepSeek V3.1 (68.5% vs 57.1%; *χ*^2^_3_=3.24; *P*=.07), and Qwen3-32B (58.2% vs 51.6%; *χ*^2^_3_=1.79; *P*=.18). These findings suggest that simply providing longitudinal full-course data did not significantly enhance the diagnostic accuracy of any of the evaluated LLMs in this EICU cohort.

### Secondary End Point 2: Number of Correct Diagnoses in the Top-3 Outputs in the Final Diagnosis Stage

**Figure 4:**
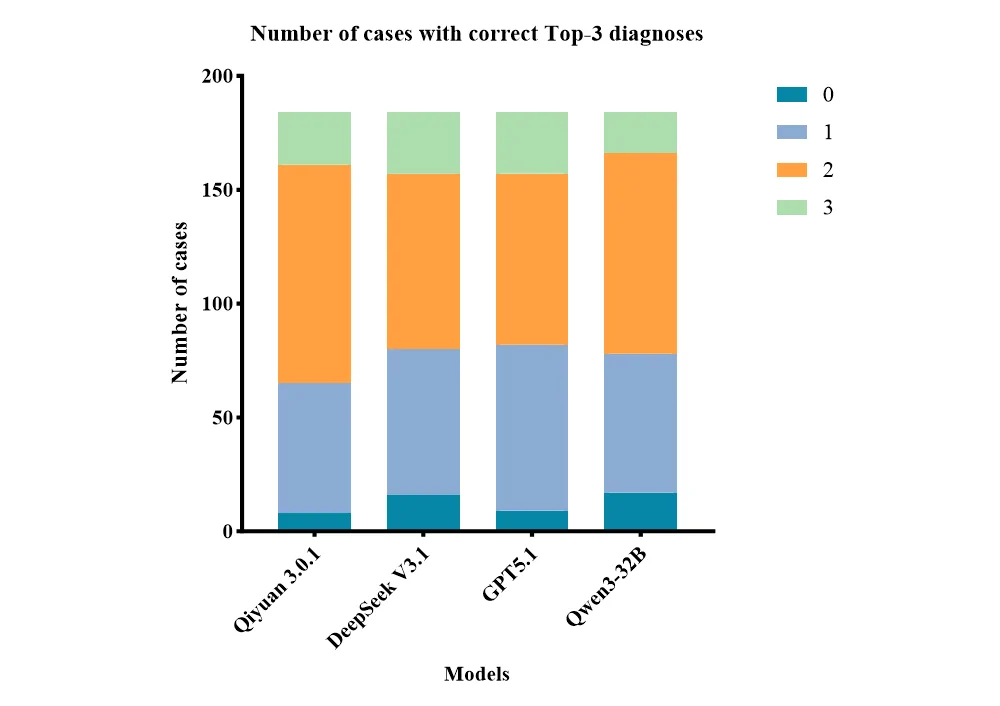
Distribution of the number of concordant diagnoses within each model’s top 3 outputs. Stacked bar chart showing the count distribution of correctly matched diagnoses (0, 1, 2, or 3 matching gold standard diagnoses) among the top 3 ranked diagnostic outputs of 4 large language models (LLMs). Data are from a single-center retrospective paired study of 184 consecutive emergency intensive care unit patients (Peking University Shenzhen Hospital, April 2025-March 2026). The x-axis represents the 4 evaluated LLMs, and the y-axis represents the absolute number of cases. Color strata indicate the number of concordant diagnoses.

The median number of correct top-3 diagnoses for all 4 models was 2.0 (IQR 1.0-2.0), with no significant overall intergroup difference (Friedman *χ*^2^_3_=3.34; *P*=.34). The 95% CIs of the indicators of each model overlapped widely, indicating that there was no significant statistical difference in the overall diagnostic performance of the 4 models at the level of multidiagnosis matching in the final diagnosis. Figure 4 illustrates the distribution of the number of concordant diagnoses among the top-3 outputs for each model.

To further explore the diagnostic differences of the models in different common EICU diseases, we performed a stratified analysis of the 5 main diseases with ≥10 cases based on the final diagnosis dataset. The results showed that there was obvious heterogeneity in the diagnostic accuracy of each model in different diseases, as shown in Table 3.

**Table 3:** Subgroup analysis of top‑1 diagnostic accuracy by major emergency intensive care unit disease categories.a

Disease	Qiyuan 3.0.1, n/N (%)	DeepSeek V3.1, n/N (%)	Qwen3-32B, n/N (%)	GPT-5.1, n/N (%)
Sepsis (n=39)	17/39 (43.6)	23/39 (59)	21/39 (53.8)	23/39 (59)
Severe pneumonia (n=36)	23/36 (63.9)	17/36 (47.2)	10/36 (27.8)	14/36 (38.9)
Gastrointestinal bleeding (n=17)	17/17 (100)	9/17 (52.9)	11/17 (64.7)	13/17 (76.5)
Cardiogenic diseases^b^ (n=24)	13/24 (54.2)	12/24 (50)	13/24 (54.2)	13/24 (54.2)
Severe pancreatitis (n=10)	10/10 (100)	10/10 (100)	8/10 (80)	10/10 (100)

^a^Data are from a single‑center retrospective paired study of 184 consecutive emergency intensive care unit patients (Peking University Shenzhen Hospital, April 2025-March 2026). Subgroup sample sizes were insufficient for formal intermodel statistical testing; results are descriptive only and should not be interpreted as evidence of model superiority or inferiority in any specific disease category.

^b^The apparent heterogeneity across disease subgroups may be influenced by small sample sizes and should be interpreted with caution. Cardiogenic diseases include acute coronary syndrome, acute heart failure, and cardiogenic shock.

## Discussion

### Summary of Key Findings

This study used a single‑center retrospective matched‑pair design to evaluate the diagnostic performance of a critical care–dedicated LLM (Qiyuan 3.0.1) vs general‑purpose models (GPT-5.1, DeepSeek V3.1, and Qwen3‑32B) at both the initial and final diagnostic stages in the EICU. Three core findings emerged. First, at the final diagnostic stage, the top‑1 accuracy across the 4 models differed significantly overall, with Qiyuan 3.0.1, GPT-5.1, and DeepSeek V3.1 all significantly outperforming Qwen3‑32B, whereas no significant differences were observed between Qiyuan 3.0.1 and GPT-5.1 or DeepSeek. Second, irrespective of the initial or final stage, the top‑1 accuracy of all models remained below 70%. Third, at the final stage, no significant differences were found among the 4 models in the number of correctly identified top‑3 diagnoses. However, the overall comparable performance warrants careful interpretation in light of disease-specific subgroup findings. In the sepsis subgroup—the largest disease category in this cohort (n=39, 21.2%)—Qiyuan 3.0.1 showed the lowest descriptive top-1 accuracy (17/39, 43.6%) among all 4 models, compared with 59% (23/39) for GPT-5.1 and DeepSeek V3.1, and 53.8% (21/39) for Qwen3-32B. This observation is clinically notable, as sepsis is one of the most prevalent and prototypical conditions in EICU practice. Conversely, the model achieved 100% accuracy in smaller subgroups such as gastrointestinal bleeding (n=17) and severe pancreatitis (n=10). These contrasting findings suggest that the performance of the critical care–specialized model may be disease-dependent rather than uniformly generalizable across all EICU conditions. Overall, under the specific conditions of our dataset, the critical care–specialized model demonstrated performance comparable to the general‑purpose models included in this study (GPT-5.1 and DeepSeek V3.1), but did not statistically significantly surpass them.

The results of this study warrant cautious interpretation. The 4 models differ systematically in parameter scale, architectural type, training corpus composition, alignment strategies, and deployment environments—all of which may be associated with model performance. Consequently, performance differences cannot be simply attributed to whether a model has undergone domain‑specific training. Specifically, Qiyuan 3.0.1 showed numerically higher final‑stage accuracy than Qwen3‑32B, a model of similar parameter scale (64.1% vs 51.6%), yet this difference must be understood in light of their differing training strategies. These patterns suggest that, under the task conditions defined in this study, larger parameter scale did not necessarily confer improved diagnostic performance, and the contribution of specialized training remains difficult to disentangle from confounders such as scale, architecture, and training data. Hence, this investigation should be regarded as a pragmatic comparison of clinically accessible tools rather than a causal demonstration of the superiority of domain‑specific fine‑tuning.

The observed between-model differences across disease subgroups should be interpreted with caution, as the sample sizes within each disease category were limited (ranging from 10 to 39 cases). Notably, Qiyuan 3.0.1’s lower descriptive accuracy in the sepsis subgroup—the largest and most clinically representative category—raises the possibility that domain-specific fine-tuning may not uniformly enhance diagnostic performance across all critical care conditions. The apparent heterogeneity across disease subgroups may reflect genuine differences in diagnostic capability, disease-specific training data coverage, or simply chance variation attributable to small sample sizes. Future studies with larger, more balanced disease cohorts are needed to determine whether these descriptive patterns reflect stable model characteristics or sampling variability. These subgroup results should therefore be regarded as hypothesis-generating rather than conclusive.

### Comparison with Existing Literature

Compared with previous research, most prior studies on LLM diagnostic performance have concentrated on general outpatient clinics, conventional emergency departments, or general ICUs [13-16], with few dedicated comparative investigations in the EICU setting—a context characterized by rapid disease evolution, multiorgan involvement, and pronounced diagnostic uncertainty. Moreover, the majority of earlier studies evaluated only a single or a few general-purpose LLMs [17-20], lacking paired comparisons between critical care–specialized and general models in the EICU environment. In addition, most previous work assessed final diagnostic ability using complete longitudinal data, without simulating the stepwise clinical reasoning process from initial admission assessment to final confirmed diagnosis. Our study adopted a 2-dataset design (initial 24-hour data and full-course final data), strictly adhered to the TRIPOD-LLM guidelines for paired diagnostic testing [21], and incorporated both top-1 accuracy and top-3 correct diagnosis counts as outcome measures, thereby providing foundational evidence for future translational research on LLM-assisted diagnosis in the EICU [24-27].

It is important to note that the observed model rankings reflect performance under a single, standardized zero-shot prompt with explicit role-framing. The extent to which these rankings generalize to other prompt formulations, including simpler nonroleplay instructions or chain-of-thought prompting, remains to be determined. Our primary objective was to evaluate each model’s baseline diagnostic ability under conditions that mirror a clinically realistic “out-of-the-box” use scenario, rather than to estimate the upper bound of performance achievable with prompt engineering.

Notably, when moving from the initial to the final dataset, no model showed improved top-1 accuracy; some even exhibited numerical declines. McNemar tests revealed no statistically significant differences between initial and final accuracies for any model (Qiyuan 3.0.1: *P*=.42; GPT-5.1: *P*=.31; DeepSeek V3.1: *P*=.07; Qwen3-32B: *P*=.18), indicating that, with the current sample size, we cannot conclude that additional clinical information impairs model reasoning. While none of these within‑model differences reached statistical significance and the numerical pattern may partly reflect sampling variability, the consistent direction of change across all 4 models invites cautious consideration of potential mechanisms to guide future research. Several speculative explanations may be considered for this counterintuitive pattern, though none can be confirmed from the present data. First, the accumulation of longitudinal clinical data may introduce information overload or noise that obscures salient diagnostic features, particularly when models are not explicitly designed for temporal reasoning. Second, LLMs may have inherent difficulty integrating temporally sequenced information across heterogeneous data sources (eg, laboratory trends, evolving imaging findings, and specialist notes) into a coherent diagnostic synthesis, a challenge that aligns with previous observations of limited temporal reasoning capacity in LLMs [28,29]. Third, the final dataset includes confirmatory test results that—although not containing explicit diagnostic summaries—may still introduce redundancy or conflicting signals that reduce model confidence. Previous studies have suggested that LLMs are sensitive to information volume and that providing full information does not always yield optimal performance [18]. The numerical fluctuations observed in our study may be attributable to sampling variability; nevertheless, this speculation requires confirmation in larger samples. If this pattern is verified in future studies with greater power, it may suggest that current LLMs still have room for improvement in integrating massive, heterogeneous, and temporally sequenced clinical data in the EICU, and that increased information does not necessarily translate into synchronous gains in diagnostic accuracy.

Based on our evaluation of baseline model performance, the appropriate role of LLMs in the EICU is as a decision‑support tool—their value lies in rapidly synthesizing information scattered across medical records and providing differential diagnostic references, rather than replacing clinician judgment. The absolute accuracy of existing models is insufficient to support independent decision‑making; the optimal usage model is a human‑machine collaborative framework characterized by “physician‑led, model‑assisted” interactions. Future efforts should focus on continuous fine‑tuning with specialized critical care corpora, exploration of multimodal information fusion architectures, and prospective human‑machine collaborative clinical trials to verify whether LLMs can substantially reduce misdiagnosis rates, shorten diagnostic time, and improve patient outcomes under this framework.

### Limitations

This study has several limitations. First, it was a single‑center retrospective design, with a disease spectrum heavily weighted toward sepsis and severe pneumonia, institution‑specific documentation practices, and EHR system architecture, all of which may affect input quality and generalizability of performance estimates. Multicenter prospective studies are needed to further validate model generalizability.

Second, this was an offline evaluation of diagnostic efficacy under controlled conditions, without inclusion of a “physician‑plus‑model” diagnostic comparator; therefore, it cannot establish whether LLMs improve clinicians’ diagnostic accuracy or patient outcomes in real EICU settings. From a translational perspective, human‑machine collaboration trials are an essential step toward clinical deployment, but systematic baseline evaluation is a necessary prerequisite. Our results provide key baseline parameters for designing such trials; specific experimental designs are detailed in the “Future Directions” section.

Third, Qiyuan 3.0.1 is a proprietary model developed by Mindray, and its architectural details, training data composition, and fine‑tuning methods are not fully disclosed owing to commercial restrictions, which limits the reproducibility of our study to some extent. However, we confirmed with the developers that no clinical data from Peking University Shenzhen Hospital EICU were used in any stage of model development—including pretraining, supervised fine‑tuning, reinforcement learning alignment, or internal validation. The training corpora consisted solely of publicly available critical care knowledge bases and deidentified clinical datasets unrelated to research institutions, thus ruling out training‑to‑evaluation data leakage. Nevertheless, the lack of transparency regarding model architecture remains a concern for reproducibility in clinical translation. Future availability of more technical details or publicly accessible model versions would facilitate more comprehensive performance assessments.

Fourth, although masked manual deidentification was performed for all confirmed diagnostic conclusions, residual diagnostic information leakage cannot be fully excluded. Certain clinical document fields—including specialist consultation notes, pathology morphology descriptions, and surgical procedure records—may carry diagnosis-informative signals that persist after redaction of explicit diagnostic labels. Of note, treatment choices and treatment outcome records were excluded from the final dataset prior to model evaluation to further minimize potential leakage from management-related documentation. This is an inherent challenge in retrospective studies using real-world clinical documentation, as these fields contain both diagnostic signals and clinically essential context. Our dataset was strictly confined to clinical information obtainable within 24 hours of admission, to simulate genuine early clinical diagnostic scenarios. Moreover, laboratory results and imaging reports are indispensable components of clinical reasoning. We partially mitigated this risk through our 2-stage (initial vs final) design, which controls the information available to models at each diagnostic stage, and through sensitivity analyses comparing strict *ICD-10* matching with semantic soft-matching (Table S1b in Multimedia Appendix 2). We acknowledge that excluding diagnosis-informative fields such as pathology descriptions would not be appropriate in this context, as these fields represent the same type of raw clinical evidence that EICU physicians use during diagnostic workup rather than preinterpreted conclusions. Nonetheless, the extent to which residual leakage may have influenced absolute accuracy estimates cannot be precisely quantified in the current design.

Fifth, because of the disease distribution in our center, sample sizes for certain conditions—for example, severe acute pancreatitis (n=10) and severe neurological emergencies (n=12)—were limited, precluding robust statistical inference. We therefore restricted analyses for these subgroups to descriptive summaries, and conclusions await verification in multicenter, larger‑sample studies.

Sixth, the choice of zero-shot prompting over alternative strategies such as few-shot learning or chain-of-thought prompting warrants discussion. We deliberately selected zero-shot prompting for 2 primary reasons. First, the intrinsic ability of each model to directly comprehend and reason over complex medical information without task-specific exemplars was itself a key dimension of interest in this comparative evaluation. Zero-shot prompting eliminates confounding factors introduced by exemplar selection or template engineering, thereby enabling a more direct assessment of the inherent diagnostic capabilities embedded in each model’s parametric knowledge. Second, zero-shot prompting better simulates real-world EICU deployment scenarios, where clinicians expect AI tools to function reliably in a “plug-and-play” manner without iterative prompt engineering. In the time-sensitive and high-stakes environment of the EICU, the “out-of-the-box” diagnostic performance of LLMs is of greater practical relevance than performance achieved after extensive prompt optimization. Nevertheless, this choice may set a lower bound on the performance achievable with optimized prompting, and our results should be interpreted as performance under a standardized zero-shot prompt rather than the maximal diagnostic capability of each model. Additionally, the role-framing language in our prompt (eg, “profound clinical attainments” and “keen diagnostic ability”) may interact differentially with models depending on their alignment training and instruction-following behavior. Different models may respond differently to such roleplay framing, which could influence relative rankings. Our results should therefore be interpreted as performance under a standardized prompt formulation—including its specific framing—rather than the maximal diagnostic capability of each model.

Seventh, while we performed an exploratory 3-tier classification of error types (Table S3a in Multimedia Appendix 2) to provide descriptive granularity beyond overall accuracy, this taxonomy was developed for descriptive characterization of error patterns rather than as a validated clinical harm severity scale. Aggregate accuracy metrics do not reflect the clinical severity of errors. A Tier 1 error (correct diagnosis ranked second or third) indicates a ranking deviation, but we did not adjudicate potential patient harm at the case level; therefore, such errors should not be interpreted as clinically minor without direct outcome assessment. Similarly, Tier 3 errors represent complete diagnostic deviation, but the potential clinical impact of each individual error was not systematically evaluated against actual patient outcomes. The descriptive nature of this classification and the absence of case-level harm adjudication should be considered when interpreting the error distributions presented in Tables S3a and S3b in Multimedia Appendix 2.

Eighth, setting the temperature parameter to 0.0 does not guarantee complete determinism for cloud-based API models. To mitigate this, we performed a stability check on 10 randomly selected cases (approximately 5% of the cohort) with 3 repeated inferences per model under the temperature=0.0 setting, all of which produced identical outputs for all 4 models. However, because the main evaluation used 1 run per case, output variability across the full cohort was not systematically characterized. This is a limitation, as output stochasticity is inherent to LLMs, and despite our efforts to control output stability via temperature settings, residual variability at the infrastructure level—such as hardware-level floating-point variation, model serving batching, or unannounced model updates—may introduce bias that is not fully captured by the limited stability check. Prior studies have highlighted the challenges that LLM randomness poses for clinical decision-making and scientific research [30,31]. A US‑based study [30] suggested that repeatability and reproducibility are not necessarily associated with diagnostic accuracy, but output variability may erode clinician trust, disrupt decision‑making, and limit clinical applicability. Our primary aim was to compare the 4 models under identical conditions rather than to estimate the absolute upper bound of any single model’s performance, and all models were subjected to the same single‑run setting; thus, the risk of systematic bias affecting relative rankings is manageable. Future work should perform multiple repeated inferences on key models and report CIs or SDs for performance metrics.

### Future Directions

It is important to acknowledge that diagnostic accuracy, while a critical performance metric, represents only 1 dimension of evaluating LLMs for high-stakes clinical deployment. Recent work has argued for a broader, output-based approach to AI alignment, proposing that LLMs should be assessed by their expressed value-priority profiles and their convergence with human judgments across dimensions such as performance, adaptive capacity, social good, ethics and responsibility, relational integration, and agency [32]. While our study focused on establishing a foundational diagnostic accuracy benchmark and did not directly assess these dimensions, this perspective highlights an important direction for future research. Even if a model demonstrates high diagnostic accuracy, its “value-in-action”—such as its prioritization of safety, transparency, or empathy—could critically influence its suitability and safety in a real-world EICU setting. Future research should therefore extend the present work by incorporating these human-value benchmarks to develop a more holistic evaluation framework for clinical LLMs, ensuring that models are not only accurate but also aligned with the ethical and relational values essential for patient care. Future research should focus on the following areas. First, multicenter external validation is urgently needed to verify the robustness of our findings in broader patient populations and clinical settings, while including general control models of comparable parameter scale to more rigorously isolate the net effect of specialized fine‑tuning. Second, prospective randomized controlled trials of human‑machine collaboration should be designed to compare “physician‑alone” vs “LLM‑assisted physician” diagnostic models in real‑world EICU settings, with end points including diagnostic accuracy, time to diagnosis, and clinical outcomes. Third, further exploration of optimization strategies tailored to the EICU context is warranted, such as fine‑tuning on critical care‑specific corpora, embedding evidence‑based diagnostic pathways, and building noise‑filtering mechanisms, with particular emphasis on improving diagnostic capability for core conditions like sepsis. In addition, prompt engineering optimization and systematic error pattern analysis represent important future directions. Specifically, future work should evaluate the stability of model rankings across multiple prompt formulations—including nonroleplay and chain-of-thought prompts—to disentangle the effects of prompt-model interaction from intrinsic diagnostic capability. Finally, establishing a standardized, publicly accessible benchmarking platform for EICU diagnostic tasks would provide uniform evaluation criteria for various models and enable fair horizontal comparisons.

### Conclusion

This study demonstrates that, within the specific data conditions of our investigation, the critical care–specialized model Qiyuan 3.0.1 performed comparably to general-purpose models (GPT-5.1 and DeepSeek V3.1) in overall top-1 accuracy. However, disease-specific subgroup analyses revealed notable heterogeneity: Qiyuan 3.0.1 showed lower descriptive accuracy in the sepsis subgroup—the largest and most clinically prototypical EICU category—while achieving high accuracy in smaller subgroups such as gastrointestinal bleeding and severe pancreatitis. These findings suggest that the potential benefit of domain-specific fine-tuning may be disease-dependent rather than uniform, and warrant further investigation in larger, more balanced cohorts. Nevertheless, the overall accuracy of all models remained below 70%, precluding their direct use as an independent diagnostic standard in clinical decision-making. The path from preliminary performance evaluation to clinical translation still requires overcoming several key hurdles: validating the robustness and generalizability of our findings in larger, multicenter datasets; designing prospective human-machine collaborative trials to determine whether model assistance genuinely improves diagnostic efficacy and patient outcomes; and pursuing deeper model optimization for critical care scenarios, including sustained fine-tuning on specialty corpora, transparent design of diagnostic reasoning pathways, and systematic assessment of clinical safety boundaries.

## Appendix

Multimedia Appendix 1 TRIPOD-LLM Checklist for Diagnostic Accuracy Studies.

Multimedia Appendix 2 Supplementary Tables S1a-S3b: summary of interrater reliability, sensitivity analysis of ICD‑10 matching vs semantic soft‑matching, model configuration details, and descriptive classification of diagnostic error types with confusion categories.

## Abbreviations

APACHE II: Acute Physiology and Chronic Health Evaluation II
API: application programming interface
EICU: emergency intensive care unit
ICD-10: International Classification of Diseases, Tenth Revision
ICU: intensive care unit
LLM: large language model
MDT: multidisciplinary team
TRIPOD-LLM: Transparent Reporting of a Multivariable Model for Individual Prognosis or Diagnosis for Large Language Models

## Data Availability

The deidentified clinical dataset, raw diagnostic output data of the large language models, and statistical analysis code of this study are available from the corresponding author upon reasonable and compliant request. Data sharing has been approved by the ethics committee.
