# Supplementary material for: Critical Care–Specific vs General-Purpose Large Language Models in Emergency Intensive Care Unit Diagnosis: Single-Center Retrospective Paired Comparative Study
Source: J Med Internet Res. 2026 Aug 11;28:e98026. doi: 10.2196/98026 (PMC13507718; doi:10.2196/98026)
Supplement: Supplementary file 2 [file jmir_v28i1e98026_app2.docx]

**Supplementary Table S1a. Summary of Inter-Rater Reliability Across All Evaluation Stages**

| Assessment Dimension | Assessment Content | Kappa Type | Kappa (95% CI) |
| --- | --- | --- | --- |
| Initial diagnosis stage | Binary classification (correct/incorrect) | Unweighted Cohen‘s Kappa | 0.92 (0.89–0.95) |
| Final diagnosis stage | Semantic flexible matching | Unweighted Cohen’s Kappa | 0.86 (0.81–0.91) |
| Top-3 final diagnosis | Ordinal scoring (0–3) | Quadratic weighted Cohen's Kappa | 0.95 (0.93–0.96) |
| Gold standard (physician consensus)* | Primary diagnosis | Fleiss' Kappa | 0.82 (0.76–0.88) |
| Gold standard (physician consensus)* | Secondary diagnoses | Fleiss' Kappa | 0.79 (0.72–0.85) |
| *Reported in Methods, Section 4 (Gold Standard Establishment). | | | |

**Legend: Cohen’s Kappa was used to evaluate inter‑rater agreement between the two independent adjudicators for model‑output scoring. Fleiss’ Kappa was used to evaluate agreement among the three senior intensivists in establishing the gold‑standard diagnoses. Values above 0.80 indicate almost perfect agreement, values between 0.61 and 0.80 indicate substantial agreement. Data were derived from a single‑center retrospective paired study of 184 consecutive EICU patients (Peking University Shenzhen Hospital, April 2025 – March 2026).**

**Supplementary Table S1b. Sensitivity analysis of Top‑1 diagnostic accuracy under strict ICD‑10 matching versus semantic soft‑match rules in the final diagnosis stage.**

| Model | Strict ICD‑10 matching (%) | Semantic soft‑match (%) | Difference (percentage points) |
| --- | --- | --- | --- |
| Qiyuan 3.0.1 | 57.1 (105/184) | 64.1 (118/184) | +7.0 |
| GPT‑5.1 | 52.2 (96/184) | 59.2 (109/184) | +7.0 |
| DeepSeek V3.1 | 51.6 (95/184) | 57.1 (105/184) | +5.5 |
| Qwen3‑32B | 46.2 (85/184) | 51.6 (95/184) | +5.4 |

**Legend: Strict ICD‑10 matching required exact subcategory‑level code matching; the semantic soft‑match rule allowed clinically equivalent terminology. Despite the absolute increase in accuracy under the soft‑match rule, the relative ranking of the four models remained unchanged under both matching rules. Data were derived from a single‑center retrospective paired study of 184 consecutive EICU patients (Peking University Shenzhen Hospital, April 2025 – March 2026).**

**Supplementary Table S2. Details of the four large language models evaluated in this study**

| Model Identifier/Version | Source or Access Method | Deployment Mode | Access/Test Date | Parameter Scale | Reasoning Mode | Generation Parameters / Compatibility |
| --- | --- | --- | --- | --- | --- | --- |
| Qiyuan 3.0.1  (release 2025.08.01) | Mindray internal model, built upon Qwen3-32B with additional domain‑specific supervised fine‑tuning and reinforcement learning optimization | On Premise | 2026‑03‑01 | 32B | Thinking mode | Temperature=0.0, Top‑p=1.0, Top‑k=0 (disabled), Max tokens=20000, Random seed=42 — all parameters effective |
| Qwen3‑32B  (release 2025.04.28) | Model weights downloaded from Hugging Face Model Hub^1^ | On Premise | 2026‑03‑01 | 32B | Thinking mode | Temperature=0.0, Top‑p=1.0, Top‑k=0 (disabled), Max tokens=20000, Random seed=42 — all parameters effective |
| GPT‑5.1  (release 2025.11.25) | Microsoft Azure OpenAI Service API^2^ | Cloud API call | 2026‑03‑01 | Undisclosed by OpenAI^3^ | Thinking mode | Temperature=0.0, Top‑p=1.0, Top‑k=0 (disabled), Max tokens=20000, Random seed=42 — all parameters effective |
| DeepSeek V3.1   (release 2025.08.21) | Volcengine / Volcano Ark API (ByteDance)^4^ | Cloud API call | 2026‑03‑01 | 685B | Thinking mode | Temperature=0.0, Top‑p=1.0, Top‑k=0 (disabled), Max tokens=20000, Random seed=42 — all parameters effective |

**Legend:**

**¹Hugging Face Model Hub:** [**https://huggingface.co/Qwen/Qwen3-32B**](https://huggingface.co/Qwen/Qwen3-32B)

**² Microsoft Azure OpenAI Service API:** [**https://azure.microsoft.com/en-us/products/ai-services/openai-service**](https://azure.microsoft.com/en-us/products/ai-services/openai-service)

**³ OpenAI has not publicly disclosed the parameter count of GPT-5.1. The official OpenAI API documentation confirms that this information is not released (see:** [**https://platform.openai.com/docs/models/gpt-5-1**](https://platform.openai.com/docs/models/gpt-5-1)**).**

**^4^** **Volcengine / Volcano Ark API (ByteDance):** [**https://www.volcengine.com/product/ark**](https://www.volcengine.com/product/ark)

**All models were accessed on the same date to control for temporal variability. Deployment modes are classified as local (on premises server deployment) or cloud based (via application programming interface [API] calls). All models used the same reasoning mode (enable_thinking=true) with standard generation. Parameter compatibility varied across deployment modes: Temperature, Top p, Top k, and Max tokens were supported by all models; the random seed parameter was supported by local deployments (Qwen3 32B and Qiyuan 3.0.1) but was either not guaranteed (Azure OpenAI API for GPT 5.1) or not supported (Volcengine API for DeepSeek V3.1) for cloud based models. Qiyuan 3.0.1 is a proprietary model developed by Mindray, built upon the open source Qwen3 32B architecture with additional domain specific supervised fine tuning and reinforcement learning optimization. *Mindray internal model, fine tuned from Qwen3 32B with reinforcement learning. All other model specifications are derived from the developers' publicly available technical documentation.**

**Supplementary Table S3a. Descriptive Classification of Diagnostic Error Types by Each Model**

| Model | Total Cases | Any Error | Tier 1 | Tier 2 | Tier 3 |
| --- | --- | --- | --- | --- | --- |
| Qiyuan 3.0.1 | 184 | 66 | 18 (27.3%) | 6 (9.1%) | 42 (63.6%) |
| GPT-5.1 | 184 | 75 | 12 (16.0%) | 15 (20.0%) | 48 (64.0%) |
| DeepSeek V3.1 | 184 | 79 | 7 (8.9%) | 16 (20.3%) | 56 (70.9%) |
| Qwen3-32B | 184 | 89 | 10 (11.2%) | 36 (40.5%) | 43 (48.3%) |

**Errors were descriptively classified into three tiers based on diagnostic distance from the gold standard, rather than validated clinical severity. Tier 1: the correct diagnosis appeared among the model‘s top-3 outputs but not in first place. Tier 2: the diagnosis fell within the correct disease category but the subcategory was wrong (e.g., “pneumonia” vs. “community-acquired pneumonia”). Tier 3: the diagnosis was fundamentally wrong in etiology or organ system (e.g., sepsis misdiagnosed as malignancy, or acute heart failure misdiagnosed as pneumonia). Percentages were calculated as the proportion of each tier among all errors for that model. Data were derived from a single-center retrospective paired study of 184 consecutive EICU patients (Peking University Shenzhen Hospital, April 2025 – March 2026).**

**Supplementary Table S3b. Dominant confusion categories for Tier 3 complete deviation errors**

| Model | Infection → Malignancy | Cardiopulmonary misclassification | CNS etiology misattribution | Superficial diagnosis | Other errors |
| --- | --- | --- | --- | --- | --- |
| Qiyuan 3.0.1 | 7 | 5 | 4 | 6 | 20 |
| GPT-5.1 | 10 | 3 | 3 | 4 | 28 |
| DeepSeek V3.1 | 7 | 4 | 3 | 25 | 17 |
| Qwen3-32B | 4 | 4 | 3 | 20 | 12 |

**This table provides a qualitative breakdown of Tier 3 errors (complete deviation in etiology or organ system) across the four models. "Superficial diagnosis" refers to assigning a non‑specific symptom complex (e.g., dizziness, weakness, or general malaise) as the primary diagnosis while missing the underlying life‑threatening etiology. "Other cross‑system errors" includes misclassifications that do not fit into the preceding four categories (e.g., metabolic disorders misdiagnosed as primary psychiatric conditions, drug toxicity or overdose misdiagnosed as infection, or renal failure misdiagnosed as hepatobiliary disease). The total number of Tier 3 errors for each model is provided in the Tier 3 column of Supplementary Table S3a. Data were derived from a single‑center retrospective paired study of 184 consecutive EICU patients (Peking University Shenzhen Hospital, April 2025 – March 2026).**
